# Stereotactic Radiosurgery for a Patient With 94 Brain Metastases in the Setting of Prior Whole Brain Radiation

**DOI:** 10.7759/cureus.86925

**Published:** 2025-06-28

**Authors:** Rituraj Upadhyay, Jonathan Schoenhals, Jayeeta Ghose, Eugene Yap, Joseph Pichler, Michael Weldon, Raj Singh, Joshua D Palmer, Wesley Zoller, Aubrie Ward, Evan Thomas, Raju Raval

**Affiliations:** 1 Radiation Oncology, Ohio State University Wexner Medical Center, Columbus, USA; 2 Radiation Oncology, Ohio State University College of Medicine, Columbus, USA

**Keywords:** brain metastases with nsclc, egfr mutant lung adenocarcinoma, linac-based radiosurgery, osimertinib, radiation therapy, whole brain rt

## Abstract

The current standard of care treatment for patients with ≥15 brain metastases (BM) is whole brain radiation therapy (WBRT), despite poor neurocognitive outcomes. Here, we present our experience treating a young patient with 94 intact brain metastases with SRS in the setting of prior WBRT. A 37-year-old male with metastatic lung adenocarcinoma (PD-L1 5%, EGFR exon 19 deletion) initially presented with a seizure and numerous intracranial metastases and previously completed a course of WBRT to a total dose of 3000 cGy in 10 fractions at an outside hospital. He subsequently started first-line oral osimertinib therapy, with baseline PET/CT showing multiple sites of disease. After 18 months from initial diagnosis and WBRT, the patient presented with 94 new brain metastases while on maintenance osimertinib. He had a Karnofsky performance score of 90, no neurological deficits, and only occasional headaches. His baseline cognitive objective Patient-Reported Outcome Measurement Information System (PROMIS) score was 29/40. Given his age, failure of EGFR-targeted therapy, and prior WBRT, he was planned for single-isocenter multiple target (SIMT) fractionated SRS to all lesions to a total dose of 2400 cGy in three fractions to 91 lesions and 1800 cGy to three brainstem metastases. He was simulated with a Qfix© Encompass mask (Qfix, Avondale, PA, USA) and treated on a Varian Edge linear accelerator utilizing HyperArc (Varian, Palo Alto, CA, USA), a 6DOF robotic couch with daily CBCT, and a Varian Optical Surface Monitoring System. A planning target volume (PTV) was created using a 2 mm margin around the GTV, with a smaller margin of 1 mm for brainstem metastases. The total GTV was 8.6 cc and PTV was 40.1 cc. He tolerated SRS well with no acute side effects. Due to progressive systemic disease, he transitioned to atezolizumab, paclitaxel, carboplatin, and bevacizumab combination therapy. Follow-up MRI imaging at two and five months was consistent with post-treatment changes, with no increase in the volume or number of brain metastases. His serial PROMIS scores were 29, 29, and 26 at three, six, and nine months of follow-up, respectively. At the last follow-up, 11 months after SRS, he remained free of headaches or new neurological symptoms. Due to the systemic progression of the disease, he transitioned to comfort care 30 months after BM diagnosis and 11 months after SRS. This case illustrates one of the largest numbers of metastases treated in a single course of SRS, and this treatment was well tolerated, with no significant cognitive decline, resulting in a comparable survival outcome to contemporary studies evaluating WBRT in this population.

## Introduction

Outcomes for patients with brain metastases have improved with advances in radiation techniques and systemic therapies, including immunotherapy [1,2]. Whole brain radiation therapy (WBRT) is the current standard of care treatment for patients with multiple brain metastases, despite several side effects, including neurocognitive decline [3]. Stereotactic radiosurgery (SRS) has emerged as a highly precise radiation modality that allows targeted treatment of multiple lesions while sparing healthy brain tissue, thus reducing cognitive risks. SRS has been shown to have excellent local tumor control with minimal side effects and is now considered the treatment of choice for patients with limited brain metastases [4,5]. As technology evolves, the threshold for using WBRT over SRS continues to shift, with ongoing trials exploring SRS in patients with 10 or more brain metastases. In addition, recent studies such as the QUARTZ trial have established the role of optimal supportive care in patients with multiple brain metastases who are unsuitable for surgical resection or SRS, with a median survival of two to six months with this approach.

One key challenge in treating multiple metastases with SRS - especially Gamma Knife based - is the extended total treatment time. However, newer LINAC-based platforms, such as HyperArc (Varian, Palo Alto, CA, USA), significantly shorten treatment time. Volumetric modulated arc therapy (VMAT), an advanced intensity-modulated radiation therapy-based plan optimization platform, enables single isocenter treatment of multiple lesions by modulating gantry rotation, collimation, and dose rate [6-8]. Several studies have demonstrated the utility of this technique in treating multiple metastases, but data are still emerging on its feasibility and safety in patients with more than 20 brain metastases [9-11]. Here, we present our experience treating a young patient with 94 new brain metastases with SRS after prior WBRT, with the objective of evaluating the safety and feasibility of treating multiple brain metastases with SRS in this setting. 

## Case presentation

A 37-year-old gentleman with no significant past medical history presented with a partial seizure involving the left upper extremity with secondary generalization. Baseline MRI brain revealed numerous bilateral cerebral and cerebellar metastases with surrounding vasogenic edema. Staging CT scan of the chest, abdomen, and pelvis showed a 1.4 cm right lower lobe lung nodule. A CT-guided biopsy of the lung nodule confirmed primary lung adenocarcinoma, with EGFR exon 19 deletion and a PD-L1 score of 5%. A whole body 18FDG-PET/CT scan revealed mediastinal nodal and osseous metastases. He completed a course of WBRT 3000 cGy in 10 daily fractions prior to presenting to us and was subsequently started on first-line therapy with oral osimertinib. Restaging imaging in six months was suggestive of a partial systemic response.

**Figure 1:**
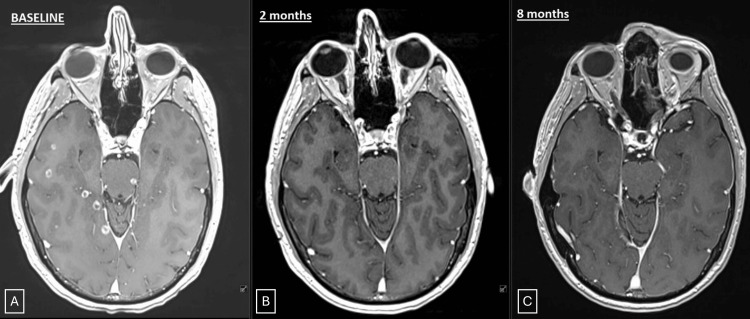
Serial contrast-enhanced MRI images. A: Axial sections of baseline contrast-enhanced MRI scan demonstrating multiple metastases, including brainstem lesions. B: Follow-up scan at two months. C: Follow-up scan at eight months.

After about 18 months since initial diagnosis and WBRT, the patient presented with >90 new brain metastases on interval MRI brain imaging, including new brainstem metastases, while still on osimertinib (Figure 1A) and was referred to our institution. He reported only occasional headaches, had no neurological deficits, and had a Karnofsky performance status of 90. His baseline Patient-Reported Outcome Measurement Information System (PROMIS) cognitive score was 29/40.

**Figure 2:**
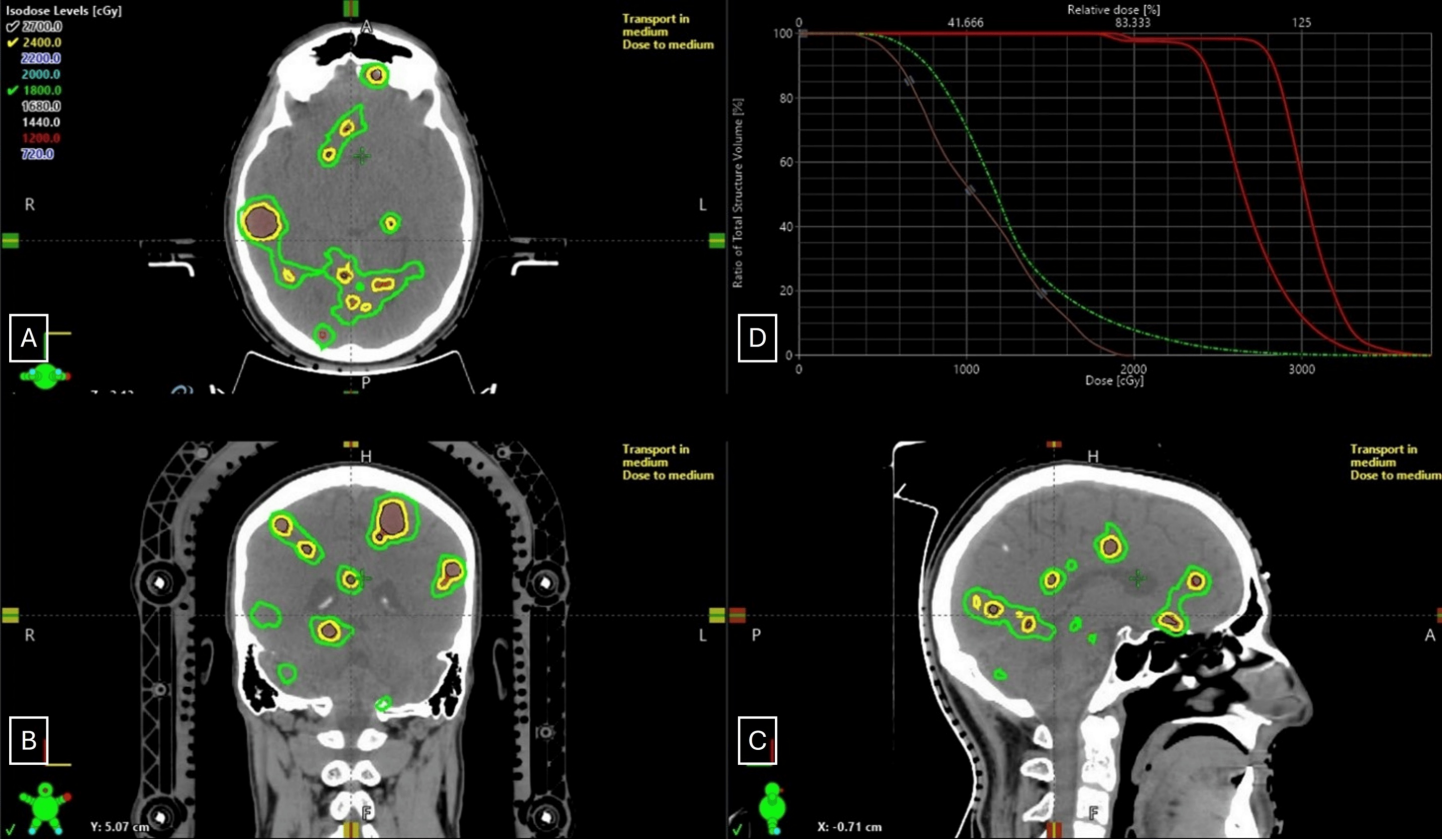
Radiation treatment plan Axial (A), coronal (B), and sagittal (C) sections representing radiation dose color wash; and dose volume histogram (D). Dose volume histogram demonstrates volumetric doses received by total planning target volume (respective PTV2400 and PTV1800 in red), whole brain (green), and brainstem (brown).

Given his young age, prior WBRT, and failure of EGFR-targeted therapy, fractionated SRS was recommended. He underwent SRS to 94 lesions: 91 treated to 2400 cGy in three daily fractions and three brainstem lesions to 1800 cGy. Simulation was performed in supine position with a Qfix© Encompass thermoplastic mask (Qfix, Avondale, PA, USA), and treatment was delivered using HyperArc technique on a Varian Edge linear accelerator (Varian, Palo Alto, CA, USA). A robotic couch with six degrees of freedom, daily kV cone-beam CT image guidance, and surface-guided radiation therapy using the Varian Optical Surface Monitoring System was used. Gross tumor volume (GTV) was defined on fused volumetric contrast-enhanced MRI. A planning target volume (PTV) was created using a 2 mm margin around the respective GTVs, with a smaller margin of 1 mm for brainstem metastases. The total GTV was 8.6 cc and PTV 40.1 cc (Figure 2). A single-isocenter multi-target VMAT plan using four noncoplanar 6 MV flattening filter-free (FFF) arcs was employed. The conformity index was 0.67, and the mean GTV dose of 3029 cGy. The total mean brain dose was 1220 cGy, and the brain V20 was 83.7 cc (Table 1). He tolerated the treatment well with no acute side effects. Osimertinib was held one day before to one day after the course of radiation therapy. 

**Table 1:** Radiation plan details

Number of lesions	94
Total GTV	8.6 cc
Total PTV	40.1 cc
GTV mean dose	30.29 Gy
GTV max dose	37.75 Gy
Conformity index	0.67
Total brain volume	1545 cc
Brain V20	83.7 cc
Brain mean dose	12.2 Gy

Concurrent systemic progression led to second-line systemic therapy with atezolizumab, paclitaxel, carboplatin, and bevacizumab. Follow-up brain MRI at two and five months was consistent with post-treatment changes, with no increase in the volume or number of brain metastases (Figure 1B). His serial PROMIS scores were 29, 29, and 26 at three, six, and nine months of follow-up, respectively. He did have a mild increase in size of some treated lesions at eight months, which were low on perfusion imaging, suggestive of grade 1 radiation necrosis (Figure 1C). At the last follow-up, 11 months after SRS, he remained neurologically intact and free of symptoms. Unfortunately, due to systemic progression of disease in the lungs and liver, he was transitioned to comfort care, approximately 30 months after initial brain metastases diagnosis.

## Discussion

Radiosurgery has advanced significantly over the past few years due to improvements in imaging techniques, radiation treatment planning, and image guidance. SRS has been traditionally used for patients with a limited number of brain metastases, but is now being increasingly used for patients with higher lesion counts [12-14]. The major benefits of SRS include excellent rates of local tumor control; minimal side effects including typically no fatigue, hair loss, or increased ICP-related symptoms leading to minimal disruption of daily routine and excellent short-term quality of life; improved neurocognitive outcomes; no delay in systemic therapy due to one to five fraction treatments; no synergistic chemoradiation or tyrosine kinase inhibitor related neuro-toxicity; and excellent results with salvage SRS. Studies have also shown that SRS can minimize neurogenic stem cell injury, which can happen with WBRT and can cause damage to normal brain tissue. However, despite the promising potential, there is limited evidence validating the use of SRS in the management of patients with multiple brain metastases, and patients with 15 or more metastases are often treated with WBRT. We present here the treatment of one of the largest numbers of metastases in a single course of SRS and show the feasibility of such an approach in a clinical setting with the aim of preserving patient neuro-cognition, while preventing neurological death.

Studies comparing SRS and WBRT have consistently reported improved cognitive outcomes with SRS over WBRT [14,15]. SRS has been shown to decrease the risk of cognitive decline by almost 30% at four months (52% vs. 24%) [5]. A recent secondary analysis of the N107C clinical trial comparing SRS and WBRT for <5 brain metastases reported that cognitive deterioration is less frequent with SRS (37-60%) compared with WBRT (75-91%) at all follow-up time points after treatment [3]. The difference in cognitive deterioration was shown to start as early as three months after RT, and in fact, the difference between SRS and WBRT was highest at three months (37% vs. 88.9%). In addition, it has also been shown that the number of metastases treated with SRS does not impact neurocognitive outcomes [15]. We did not observe any cognitive decline after SRS in our patient per the NIH PROMIS score [16]. Given these, patients with multiple brain metastases live longer with the improvements in systemic therapies, especially in the setting of driver mutations; they are expected to survive long enough to benefit from the cognitive sparing effects of SRS over WBRT.

While WBRT may offer better intracranial control, it does not provide any additional survival benefit [4]. The total intracranial control at one year in the secondary analyses of N107C was 40.7% with SRS alone versus 81.5% with WBRT (p = 0.003). However, recurrences post-SRS can often be salvaged with repeat SRS.

Our study has certain limitations, including generalizability from a single case and potential selection bias. Many recent and ongoing clinical trials focus on using an arbitrary number of lesions as a criterion for SRS eligibility. Ultimately, volume-based thresholds rather than lesion count may best guide treatment decisions. Studies suggest that total tumor volume, rather than the absolute number of lesions, is a more reliable predictor of outcomes [17-22]. In our case, despite treating 94 lesions, the total GTV was only 8.6 cc, comparable to or lower than many series reporting favorable outcomes. Studies by Likhacheva et al. and Kim et al. reported poorer outcomes when the cumulative tumor volume was greater than 2-7 cc [18,21]. A recent study by the MAASTRO group in the Netherlands compared SRS (15-24 Gy in a single fraction) with WBRT for patients with four to 10 metastases, limiting inclusion to lesions no larger than 2.5 cm in diameter and a total cumulative tumor volume of 30 cm^3^. They reported a one-year overall survival of 57% with SRS versus 31% with WBRT. In addition, patients treated with SRS maintained a consistently better quality of life compared to those who received WBRT [23,24]. The total tumor volume in our patient was well less than the upper limit of this study. Ongoing trials like NAGKC 12-01 (NCT01731704) are further exploring SRS in patients with ≥5 metastases and ≤15 cc total volume [25].

## Conclusions

We present here the treatment of one of the largest numbers of metastases in a single course of SRS. Although this is a single case report, this case illustrates the feasibility and safety of frameless, LINAC-based SRS using a single-isocenter VMAT technique in a patient with over 90 brain metastases. This permits non-invasive, fast, and accurate targeting of multiple metastases simultaneously. As imaging and systemic therapies improve, long-term survival in this patient population is increasing, making cognitive preservation paramount. Our experience supports the growing evidence that SRS is a viable alternative to WBRT in selected patients with a high number of brain metastases but a low total tumor volume. Our study has potential implications for advancing clinical guidelines advocating SRS based on total metastatic volume rather than number of metastases, and informing future trial designs exploring cognitive outcomes with SRS versus WBRT.
